# Tumor Cell Proportion Assessment in Advanced Non-Squamous Non-Small Cell Lung Cancer Tissue Samples in Real-World Settings in Japan: The ASTRAL Study

**DOI:** 10.3390/diagnostics15172165

**Published:** 2025-08-26

**Authors:** Kanako C. Hatanaka, Kazumi Nishino, Tomoyuki Yokose, Hiroshi Tanaka, Noriko Motoi, Kenichi Taguchi, Yoichi Tamai, Takehiro Hirai, Yutaka Yabuki, Yutaka Hatanaka

**Affiliations:** 1 Center for Development of Advanced Diagnostics, Hokkaido University Hospital, Sapporo 060-8648, Japan; kyanack@huhp.hokudai.ac.jp; 2 Department of Thoracic Oncology, Osaka International Cancer Institute, Osaka 541-8567, Japan; 3 Department of Pathology, Odawara Municipal Hospital, Odawara 250-8558, Japan; 4 Department of Internal Medicine, Niigata Cancer Center Hospital, Niigata 951-8566, Japan; 5 Department of Pathology, Saitama Cancer Center, Saitama 362-0806, Japan; 6 Department of Pathology, National Hospital Organization, Kyushu Cancer Center, Fukuoka 811-1395, Japan; 7 AstraZeneca K.K., Osaka 530-0011, Japan

**Keywords:** advanced non-squamous non-small cell lung cancer, artificial intelligence algorithm, biomarker testing, intraclass correlation coefficient, tumor cell proportion assessment

## Abstract

**Background/Objectives**: Identification of driver gene alterations helps determine first-line treatment for non-squamous non-small cell lung cancer (NSCLC). Precise assessment of tumor cell proportion is critical for accurate detection of gene alterations. ASTRAL was a multicenter, prospective, observational study to investigate the agreement in tumor cell proportion assessments between different raters. **Methods**: Tissues collected in daily clinical practice from patients with advanced NSCLC were used. Raters included local pathologists, a Central Pathology Committee (CPC), and an artificial intelligence (AI) algorithm. Hematoxylin and eosin-stained slides were assessed by local pathologists, and digitized images of those slides were assessed by the CPC and the AI algorithm. The primary endpoint was agreement in assessment of tumor cell proportion between local pathologists and the CPC, as determined using the intraclass correlation coefficient (ICC). Secondary endpoints included agreement between the AI algorithm and local pathologists or the CPC. **Results**: Tissue samples from 204 patients were assessed. The ICC for local pathologists vs. the CPC showed poor to moderate agreement (0.588 [95% confidence interval (CI) 0.483–0.674]). The AI algorithm showed moderate agreement with the CPC (ICC 0.652 [95% CI 0.548–0.733]), and poor to moderate agreement with local pathologists (ICC 0.465 [95% CI 0.279–0.604]). **Conclusions**: The ICC for the AI algorithm vs. the CPC was numerically highest among the rater pairs, indicating a level of usefulness for the algorithm. Continued efforts are needed to ensure the accurate estimation of tumor cell proportion. Integration of AI algorithms in real-world practice may contribute to this.

## 1. Introduction

First-line therapy for patients with advanced or recurrent non-squamous non-small cell lung cancer (NSCLC) is determined using molecular diagnostic testing to detect driver gene alterations [1,2]. The Japan Lung Cancer Society’s Guidelines for Diagnosis and Treatment of Lung Cancer 2024 recommend biomarker testing for nine gene alterations (epidermal growth factor receptor [*EGFR*] mutation; anaplastic lymphoma kinase [*ALK*] fusion; ROS proto-oncogene 1 [*ROS1*] fusion; mesenchymal–epithelial transition [*MET*] exon 14 skipping mutation; v-raf murine sarcoma viral oncogene homolog B1 [*BRAF*] V600E mutation; rearranged during transfection [*RET*] fusion; v-Ki-ras2 Kirsten rat sarcoma viral oncogene homolog [*KRAS*] G12C mutation; human epidermal growth factor receptor-2 [*HER2*] mutation; and neurotrophic tyrosine receptor kinase [*NTRK*] fusion) to aid in the selection of first-line treatment for these patients [2]. Both multiplex and singleplex testing can be used to detect genetic biomarkers [3], although multiplex testing is more useful as it can evaluate multiple gene alterations in a single assay using the same tumor sample. It also requires a smaller tissue sample and reduces the time taken to determine a patient’s biomarker status compared with a sequential singleplex testing approach. Thus, multiplex testing is widely used clinically. However, higher-quality samples are required for multiplex vs. singleplex testing [4].

To achieve accurate detection of gene alterations, it is critical to obtain a precise assessment of the tumor cell proportion in tissue samples [5]. For example, multiplex testing generally requires a minimum tumor cell proportion of 20% to 30% to ensure adequate sensitivity for the detection of gene alterations. However, tumor cell proportion assessment varies among pathologists, and there is a reported tendency for overestimation, which may lead to false-negative results [6,7,8]. Accurate estimations of tumor cell proportion can be difficult to obtain. When assessing hematoxylin and eosin (H&E)-stained tumor tissues, pathologists mark the test area intended for nucleic acid extraction and assess the tissue volume (total number of nucleated cells) and tumor cell proportion (proportion of tumor cells among the total nucleated cells) [4]. However, clinical specimens contain various components (e.g., inflammatory cells, fibrosis, crush, and necrosis), making it difficult to accurately distinguish and count each cell type.

Artificial intelligence (AI) algorithms aid in various aspects of healthcare, including pathological diagnosis [9]. NEC Corporation (Tokyo, Japan) has developed a machine learning algorithm to detect tissue and cell features and to make quantitative assessments of key structures in digitized images of H&E-stained tissue sections. This algorithm can calculate tumor cell proportions and may be useful for determining whether tissue samples are suitable for biomarker testing [10,11].

The ASTRAL study aimed to investigate the agreement in tumor cell proportion assessments between local pathologists, a Central Pathology Committee, and the NEC AI algorithm, using tissue samples obtained from patients with advanced non-squamous NSCLC for biomarker testing in daily clinical practice in Japan.

## 2. Materials and Methods

### 2.1. Patients and Tissue Specimens

Patients were eligible for inclusion if they were aged ≥20 years, had pathologically confirmed non-squamous NSCLC, had untreated advanced or recurrent NSCLC, had received a tumor cell proportion assessment on a formalin-fixed paraffin-embedded sample, and had undergone biomarker testing prior to initiating first-line treatment. There were no exclusion criteria.

Tumor tissue specimens must have been obtained by biopsy or surgical resection (except cell block); H&E-stained specimens must have been prepared from the same continuous section as the unstained formalin-fixed paraffin-embedded specimens that were submitted for biomarker testing. Acceptable biomarker testing was limited to the following approved companion diagnostics: Oncomine^™^ Dx Target Test Multi CDx System (Oncomine DxTT; Thermo Fisher Scientific, Waltham, MA, USA), AmoyDx^®^ Pan Lung Cancer PCR Panel (AmoyDx PLC; Amoy Diagnostics, Xiamen, China), cobas^®^ EGFR Mutation Test (Roche Diagnostics, Indianapolis, IN, USA), and the Archer^®^MET Companion Diagnostic System (Integrated DNA Technologies, Boulder, CO, USA). The Oncomine DxTT and AmoyDx PLC tested for *EGFR* mutation, *ALK* fusion, *ROS1* fusion, *BRAF* mutation, *RET* fusion, *MET* exon 14 skipping, *KRAS* mutation, *HER2* mutation, and *NTRK* fusion. The cobas test only evaluated *EGFR* mutation, and the Archer test only evaluated *MET* exon 14 skipping. All specimens were submitted for biomarker testing within 3 years from the date of sample collection.

### 2.2. Study Design

This was a multicenter, prospective, observational study conducted in Japan to investigate the agreement of tumor cell proportion assessments among local pathologists at each site, a Central Pathology Committee, and an AI algorithm in patients with newly diagnosed locally advanced, metastatic, or recurrent non-squamous NSCLC. All patients who were eligible for study inclusion were consecutively enrolled. The H&E-stained slides in which each local pathologist assessed tumor cell proportion were submitted to a scanning center, where all the slides were digitized into whole slide image files at 40× magnification using a NanoZoomer S360 Digital slide scanner (Hamamatsu Photonics K.K., Shizuoka, Japan). H&E staining of tissue sections was performed at each participating institution according to their routine protocols.

This study was performed in accordance with the Declaration of Helsinki and the Ethical Guidelines for Medical and Biological Research Involving Human Subjects and was approved by the NPO-MINS Institutional Review Board (approval number: 210237) and the Osaka International Cancer Institute Ethical Review Board (approval number: 21212). All patients provided written informed consent for study inclusion. This study was registered with the Japan Registry of Clinical Trials (registration number: jRCT1030210622).

### 2.3. Data Collection

#### 2.3.1. Participating Study Sites

Data from participating study sites, including patient characteristics, tissue sample information with tumor cell proportion, and biomarker testing information, were collected prospectively using electronic case report forms. Tumor cell proportion was assessed by each local pathologist using the H&E-stained slides and reported in 5% increments if the proportion was <50% and 10% increments if the proportion was ≥50%.

#### 2.3.2. Central Pathology Committee

The Central Pathology Committee comprised three Japanese Society of Pathology board-certified molecular pathologists who were highly skilled in the assessment of lung cancer tissue samples for biomarker testing. Tumor cell proportion was assessed independently by each central pathologist using the whole slide image files, with no other information provided; results were reported in 5% increments if the proportion was <50% and 10% increments if the proportion was ≥50%. Pathological findings, which could influence the tumor cell proportion assessment, were also graded as follows: (1) inflammatory cells (none or mild, <10%; moderate, 10–70%; severe, >70%), (2) fibrosis (none or mild, <33%; moderate, 33–66%; severe, >66%), (3) mucus (none or mild, <33%; moderate, 33–66%; severe, >66%), (4) necrosis (none or mild, <33%; moderate, 33–66%; severe, >66%), and (5) crush (none or mild, 0–<3 crushed foci that have a minor impact on the overall assessment; moderate, ≥3 crushed foci that have a minor impact on the overall assessment; severe, crushed foci that have a major impact on the overall assessment). All three central pathologists performed their assessments using NDP.serve3 software (version 3.3.50; Hamamatsu Photonics K.K.) and the same computer monitor model (Dell 23 Monitor P2319H; Dell Technologies Inc., Round Rock, TX, USA), which satisfies the requirements specified in the Digital Pathology System Technical Standard for Pathological Diagnosis, 3rd edition, published by the Japanese Society of Pathology [12]. On completion, a consensus conference was held to confirm the results of each assessment of tumor cell proportion and grade of pathological findings. Specimen assessments in which all three central pathologists were in complete agreement were adopted as the final assessment. If there were differences among the three central pathologists in the assessment value of tumor cell proportions or pathological findings, they reviewed the slides together and discussed the assessment until they reached an agreement.

#### 2.3.3. AI Algorithm

The AI algorithm was developed by NEC Corporation and can detect nucleated cells within the area of interest, distinguish tumor cells from non-tumor cells, and calculate the tumor cell proportion based on the number of each cell type. The details of the AI algorithm used in this study have been published [10,11]. Briefly, the algorithm was based on the fully convolutional deep learning approach and uses the U-Net neural network architecture, which allows for simultaneous execution of cell detection and tumor/non-tumor classification tasks, effectively reducing computational time [13]. The model was trained on more than 212,000 annotated cells, with tumor cells and non-tumor cells roughly balanced, sourced from H&E-stained tissue samples at four independent hospitals. A variety of data augmentation techniques were used for training to ensure adequate performance on specimens from institutions other than the training data source. By employing hospital-wise cross-validation, the model demonstrated robust performance and generalizability, maintaining high accuracy across different sample origins and imaging conditions [11]. The AI algorithm has the capability to calculate the number of tumor and non-tumor cells and determine tumor content with a precision of within 1% and quantify the surface area of the tissue section selected by the user, which enables calculation of the required number of unstained slides based on the chosen biomarker tests. In this study, the AI algorithm was used to assess tumor cell proportions in whole slide images, reported in 1% increments, which were then categorized into the same levels used by the other raters. There were no adjustments of brightness or color.

### 2.4. Endpoints

The primary endpoint was the agreement in assessment of tumor cell proportion between local pathologists and the Central Pathology Committee as determined using the intraclass correlation coefficient (ICC). The secondary endpoints were the agreement in assessment of tumor cell proportion between the AI algorithm and either the Central Pathology Committee or the local pathologists assessed using the ICC, the success rate of the Oncomine DxTT and AmoyDx PLC panels, and the proportions of each gene alteration.

### 2.5. Statistical Analysis

The sample size was determined on a precision basis. A sample size of 218 would yield a 95% confidence interval (CI) with a width of <20% for ≥0.5 of a plausible range of the ICC; a sample size of 140 would yield a 95% CI with a width of <25% for ≥0.5 of a plausible range of the ICC [14]. The number of targeted patients was set at 155 (minimum) to 240 (maximum) patients, assuming a 10% dropout rate.

The two study assessment populations were the full analysis set (FAS), which included all patients who met the eligibility criteria, and the tumor cell proportion analysis set (TCP), which included all patients in the FAS who had whole slide images of an acceptable quality for assessment (samples for which the proportion of analysis object area was ≥90% on the whole slide image as determined by the AI algorithm) and who had tumor cell proportion assessment values by all raters.

The ICC (A,1) for Case 3A for evaluating the degree of absolute agreement for a single measurement used in this study was based on a two-way mixed effects model that included raters as a fixed effect and patients as a random effect without interaction between them, as defined by McGraw and Wong [15]. The ICC was calculated to assess the inter-rater agreement (local pathologists vs. Central Pathology Committee) in the assessment of tumor cell proportion; 95% CIs were also reported. Local pathologists comprised pathologists at each study site and were regarded as a single rater in the analysis. Inter-rater agreement (AI algorithm vs. Central Pathology Committee and AI algorithm vs. local pathologists) was also evaluated using ICC analysis. The following categories were used to describe the magnitude of difference between raters in the assessment of tumor cell proportions: no or slight difference (absolute difference between raters of ≤10%), moderate difference (difference between 11% and 20%), or considerable difference (difference >20%). Oncomine DxTT or AmoyDx PLC success rates were defined as the percentage of samples that tested positive/negative for all five biomarkers (Oncomine DxTT: *EGFR* and *BRAF* [DNA-derived] and *ALK*, *ROS1*, and *RET* [RNA-derived]; AmoyDx PLC: *EGFR* and *BRAF* [DNA-derived] and *ALK*, *ROS1*, and *MET* [RNA-derived]).

An additional exploratory analysis using ICC to investigate the agreement in assessment of tumor cell proportion among the three rater groups (local pathologists vs. Central Pathology Committee, Central Pathology Committee vs. AI algorithm, and AI algorithm vs. local pathologists) by sampling method (surgical resection, bronchoscope biopsy, computed tomography-guided needle biopsy, echo-guided needle biopsy, or other) was also conducted.

The data were analyzed descriptively; categorical variables are summarized using frequency and proportion, and continuous variables are summarized using descriptive statistics. Missing data were not imputed. Data were analyzed using SAS^®^ version 9.4 (SAS Institute, Cary, NC, USA).

## 3. Results

### 3.1. Patients

This study enrolled 215 patients from 11 sites in Japan between March 2022 and February 2023. Of those, 209 patients were included in the FAS (six were excluded because of a violation of eligibility). Among those included in the FAS, 204 were included in the TCP (five were excluded because of a lack of whole slide images).

Patient characteristics for the FAS and TCP are shown in Table 1. The median (range) age was 70.0 (36.0–90.0) years, most patients were male (FAS 136/209 [65.1%]), and most were former smokers (FAS 117/209 [56.0%]). In the FAS, 189/209 (90.4%) patients had adenocarcinoma, and the most common Eastern Cooperative Oncology Group performance status scores were 1 and 0 (106 [50.7%] and 83 [39.7%], respectively). Most patients had stage IV disease, including 119 (56.9%) with stage IVB and 67 (32.1%) with stage IVA.

### 3.2. Tissue Sampling

Table 2 shows the tissue sampling location and method in the TCP population. Most samples were obtained from the primary tumor (153/204, 75.0%) and were collected by bronchoscope biopsy (157/204, 77.0%). Fixation in 10% neutral buffered formalin was reported for 202/204 (99.0%) samples, and the fixation time was 12–<24 h for 177/204 (86.8%) samples. The mean (standard deviation) sample thickness was 5.00 μm (0.666 μm), the median number of slides submitted for biomarker testing was 10.0 (range, 4.0–30.0), and the median estimated sample volume was 1.39 mm^3^ (range, 0.06–39.51 mm^3^).

### 3.3. Pathological Findings

Pathological findings from whole slide image analysis, as assessed by the Central Pathology Committee, are shown in Table 3. The presence of inflammatory cells was classified as moderate in 135/204 samples (66.2%) and severe in 32/204 (15.7%), indicating that inflammatory cells were present in the majority of samples. Most samples (142/204 [69.6%]) had no or mild fibrosis, although moderate and severe fibrosis were observed in 56/204 (27.5%) and 6/204 (2.9%) samples, respectively. Findings for mucus, necrosis, and crush were mostly none or mild (199/204 [97.5%], 192/204 [94.1%], and 182/204 [89.2%], respectively), with very few samples classified as moderate or severe.

### 3.4. Tumor Cell Proportions

**Figure 1:**
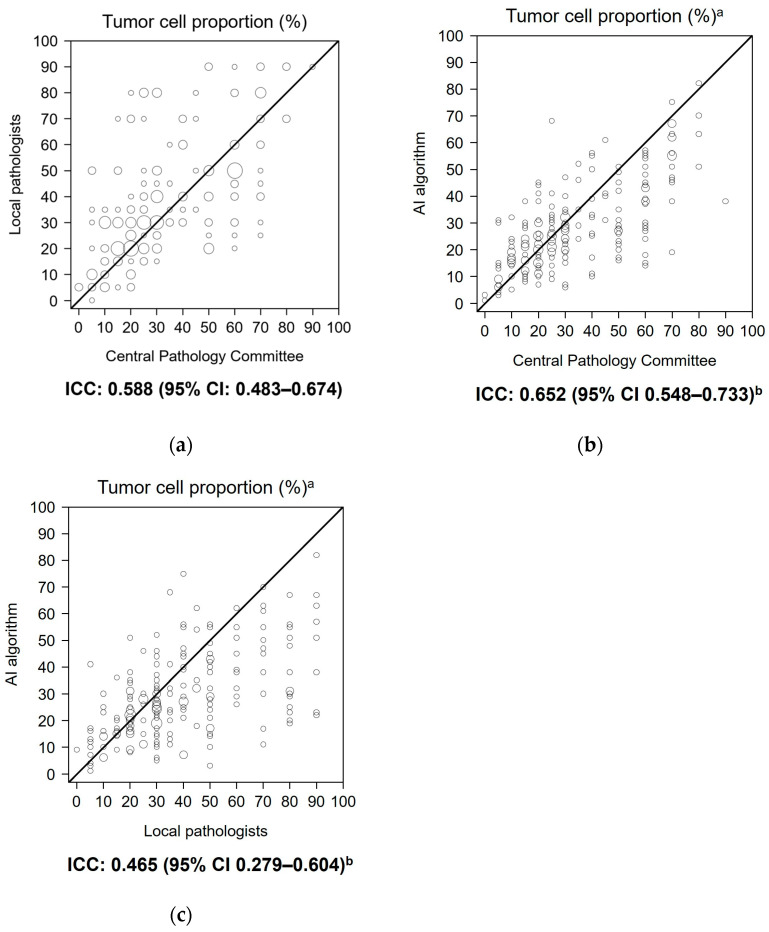
Bubble plot of tumor cell proportions for rater pairs (tumor cell proportion analysis set; *n* = 204). (**a**) Local pathologists vs. Central Pathology Committee, (**b**) AI algorithm vs. Central Pathology Committee, (**c**) AI algorithm vs. local pathologists. ^a^ The original values (1% increments) for AI-assessed tumor cell proportion are plotted. ^b^ For the ICC calculation, categorized values (5% or 10% increments) were used as the AI-assessed tumor cell proportion. Abbreviations: AI, artificial intelligence; CI, confidence interval; ICC, intraclass correlation coefficient.

The ICC for the primary endpoint (local pathologists vs. Central Pathology Committee) was 0.588 (95% CI 0.483–0.674) (Figure 1a). For the secondary endpoints, the AI algorithm vs. the Central Pathology Committee had an ICC of 0.652 (95% CI 0.548–0.733) (Figure 1b), and the AI algorithm vs. local pathologists had an ICC of 0.465 (95% CI 0.279–0.604) (Figure 1c). ICC values were not notably different among the different sampling methods in the additional exploratory analysis. For all sampling methods, the ICC values between the AI algorithm and the Central Pathology Committee were numerically highest among the pairs (Table 4).

The magnitude of differences in assessment of tumor cell proportions between raters is shown in Table 5. When comparing tumor cell proportion determinations between local pathologists and the Central Pathology Committee, there were considerable, moderate, and no or slight differences in 41 (20.1%), 38 (18.6%), and 125 (61.3%) of the 204 samples, respectively. There were considerable, moderate, and no or slight differences in 35 (17.2%), 35 (17.2%), and 134 (65.7%) of the 204 samples, respectively, when comparing the findings of the AI algorithm with the Central Pathology Committee, and 45 (22.1%), 44 (21.6%), and 115 (56.4%) samples, respectively, when comparing the findings of the AI algorithm with those of local pathologists. Compared with the Central Pathology Committee, assessments of tumor cell proportion by local pathologists and the AI algorithm were overestimated in 102 (50.0%) and 64 (31.4%) of the 204 samples, respectively; in complete agreement in 37 (18.1%) and 39 (19.1%) samples, respectively; and underestimated in 65 (31.9%) and 101 (49.5%) samples, respectively.

**Figure 2:**
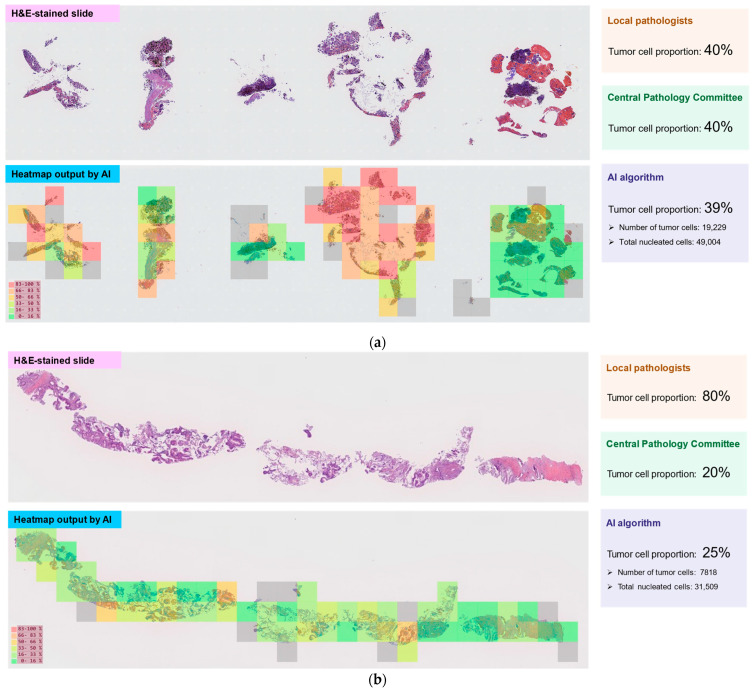

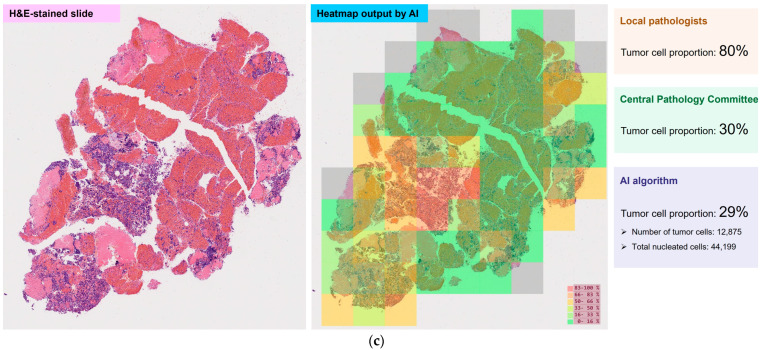
Examples of H&E-stained slide images and heatmap outputs by AI for three of the cases included in this study. (**a**) Example of tumor cell proportion agreement. Lymph node tissue samples were obtained using EBUS-TBNA from a 79-year-old female, a past smoker, with stage IVA adenocarcinoma positive for an *EGFR* mutation (test method: Oncomine™ Dx Target Test Multi CDx System). (**b**) Example of tumor cell proportion disagreement. Primary tumor tissue samples were obtained using CT-guided needle biopsy from an 87-year-old female, a never smoker, with stage IVB adenocarcinoma. No driver gene alteration was detected (test method: Oncomine™ Dx Target Test Multi CDx System). (**c**) Example of tumor cell proportion disagreement. Lymph node tissue samples were obtained using EBUS-TBNA from a 54-year-old male, a past smoker, with stage IVB large cell neuroendocrine carcinoma. No driver gene alteration was detected (test method: Oncomine™ Dx Target Test Multi CDx System). The gray shading in the heatmap output images shows the space outside the AI analysis target area. Abbreviations: CT, computed tomography; *EGFR*, epidermal growth factor receptor gene.

Figure 2 provides examples of H&E-stained slide images and heatmap outputs by the AI algorithm for three of the cases included in this study. Figure 2a shows slide images from a 79-year-old female with stage IVA adenocarcinoma. For this case, the agreement for tumor cell proportion was good among all raters (local pathologists, 40%; Central Pathology Committee, 40%; AI algorithm, 39%). In contrast, Figure 2b shows slide images from an 87-year-old female with stage IVB adenocarcinoma in which local pathologists overestimated the tumor cell proportion (80%) compared with both the Central Pathology Committee and AI algorithm (20% and 25%, respectively). Figure 2c shows slide images from a 54-year-old male with stage IVB large cell neuroendocrine carcinoma, in which local pathologists also overestimated the tumor cell proportion (80%) compared with both the Central Pathology Committee and the AI algorithm (30% and 29%, respectively).

### 3.5. Biomarker Testing

**Figure 3:**
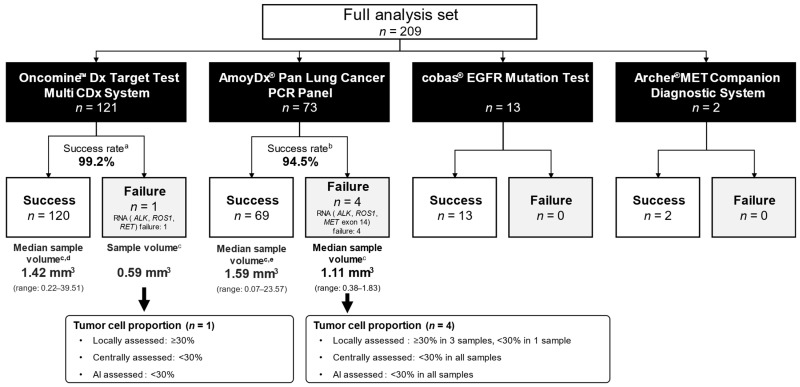
Success rate of each biomarker test (full analysis set; *n* = 209). ^a^ Defined as the percentage of samples with tested positive/negative for five biomarkers (*EGFR*, *ALK*, *ROS1*, *BRAF*, and *RET*) in samples tested by Oncomine™ Dx Target Test Multi CDx System. ^b^ Defined as the percentage of samples with tested positive/negative for five biomarkers (*EGFR*, *ALK*, *ROS1*, *BRAF*, and *MET*) in samples tested by AmoyDx^®^ Pan Lung Cancer PCR Panel. ^c^ Estimated sample volume = [thickness of formalin-fixed paraffin-embedded sections] × [number of slides submitted for biomarker testing] × [surface area of section]. ^d^ *n* = 119. One sample was not included in the median sample volume calculation because it had no data from the AI algorithm. ^e^ *n* = 66. Three samples were not included in the median sample volume calculation because they had no data from the AI algorithm. Abbreviations: *ALK*, anaplastic lymphoma kinase; *BRAF*, v-raf murine sarcoma viral oncogene homolog B1; *MET*, mesenchymal–epithelial transition; *RET*, rearranged during transfection; *ROS1*, ROS proto-oncogene 1.

Of the 209 patients in the FAS, Oncomine DxTT testing was used for 121 samples, AmoyDx PLC testing for 73 samples, cobas EGFR testing for 13 samples, and ArcherMET testing for two samples, with success rates of 99.2%, 94.5%, 100%, and 100%, respectively (Figure 3). For all samples with unsuccessful test results, failure of RNA analysis was reported. Among samples with an Oncomine DxTT test, the median (range) sample volume was 1.42 mm^3^ (0.22–39.51 mm^3^) among the 119 successful tests with AI algorithm data available and 0.59 mm^3^ for the single unsuccessful test. Among samples tested with the AmoyDx PLC, the median (range) sample volume was 1.59 mm^3^ (0.07–23.57 mm^3^) among the 66 successful tests with AI algorithm data available and 1.11 mm^3^ (0.38–1.83 mm^3^) among the four unsuccessful tests. For all five samples with unsuccessful tests, both the AI algorithm and the Central Pathology Committee assessed the tumor cell proportions as <30%, whereas the local pathologists assessed them as ≥30% in 4/5 samples.

The proportions of samples that tested positive for each gene alteration are shown in Table 6. The frequencies of *EGFR* mutation, *ALK* fusion, *ROS1* fusion, *BRAF* mutation, *RET* fusion, *MET* exon 14 skipping, *KRAS* mutation, *HER2* mutation, and *NTRK* fusion were 28.9%, 1.7%, 0.0%, 0.0%, 0.8%, 6.1%, 5.2%, 1.7%, and 0.0%, respectively, among the samples tested using the Oncomine DxTT panel; and 28.8%, 4.3%, 1.4%, 2.7%, 0.0%, 1.4%, 8.8%, 1.5%, and 0.0%, respectively, among the samples tested using the AmoyDx PLC assay. The frequency of *EGFR* mutation was 38.5% with the cobas EGFR test, and the frequency of *MET* exon 14 skipping was 0.0% with the ArcherMET test.

## 4. Discussion

This study reports ICC values of 0.588 (95% CI 0.483–0.674), 0.652 (95% CI 0.548–0.733), and 0.465 (95% CI 0.279–0.604) for local pathologists vs. Central Pathology Committee (primary endpoint), AI algorithm vs. Central Pathology Committee (secondary endpoint), and AI algorithm vs. local pathologists (secondary endpoint), respectively. Interpretation of the ICC values [16] indicates that the respective agreements were poor to moderate, moderate, and poor to moderate. The agreement in the assessment of tumor cell proportion between local pathologists and the Central Pathology Committee was not satisfactory and was numerically lower than the agreement between the AI algorithm and the Central Pathology Committee, indicating a level of usefulness for the algorithm. The exploratory analysis found no notable differences in ICC among the different sampling methods; regardless of sampling method, the ICCs between the AI algorithm and the Central Pathology Committee were numerically highest among the pairs.

Mikubo et al. have previously reported that pathologist training, in which sample images of tissues, tumor cell proportion assessments by highly-experienced pathologists, estimations by mutant allele frequencies, and detailed instructions for assessment were provided, was useful for improving the accuracy of tumor cell proportion assessment [8]. In contrast, Kazdal et al. reported that the agreement of tumor cell proportion assessment among human raters was low regardless of their level of pathological experience [17]. Furthermore, they reported the usefulness of digital pathology software for tumor cell proportion assessment, where the agreement of tumor cell proportion assessment between two software tools was higher than that within a group of 19 human raters. Carretero-Barrio et al. have reported that the reliability between pathologists in the assessment of tumor cell proportion in NSCLC whole slide images using digital image analysis was low. Although interobserver reliability slightly improved after training (ICC increased from 0.09 to 0.24), it remained poor. Subjective tasks, such as annotation, were the source of most discrepancies [18]. Kiyuna et al. reported the utility of the AI algorithm used in the present study in the assessment of tumor cell proportion in 41 regions from 41 lung cancer cases collected from multiple hospitals [11]. The authors established a ‘gold standard’ tumor cell proportion in those regions and then compared the accuracy of the tumor cell proportion as estimated by 13 pathologists based on visual assessment and as calculated by the AI algorithm. The results showed that the error between the gold standard and the AI algorithm was significantly smaller than that between the gold standard and the pathologists’ visual assessment. The study also found that the robustness against staining variations across different sites was better for the AI algorithm than the visual estimation by pathologists.

In this study, the local pathologists tended to overestimate the tumor cell proportion compared with the Central Pathology Committee, which is in agreement with previously published reports [6,7,8]. A plausible explanation is that pathologists frequently estimate tumor cell proportion by evaluating the fraction of tissue area occupied by tumor cells, rather than by quantifying individual cell counts. Given that tumor cells are typically larger than non-tumor cells, such as lymphocytes or stromal cells, this method may systematically overestimate the actual tumor cell proportion. It has been reported that NSCLC tumor tissue is characterized by the infiltration of diverse leukocyte populations [19], which may further complicate estimations. In contrast, the estimates provided by the Central Pathology Committee and the AI algorithm may offer more accurate assessments of the tumor cell proportion for the following reasons. For the Central Pathology Committee, tumor cell proportion was determined through consensus by three highly-experienced board-certified pathologists, which helps to mitigate individual variability and subjective bias. For the AI algorithm, tumor cell proportion was calculated objectively by detecting individual nuclei within the tissue section, thereby providing an estimate that is directly based on cell count rather than visual assessment of tissue area. Overestimation of tumor cell proportion in a sample can reduce the accuracy of biomarker results if the actual tumor cell proportion is below the limit of detection for a specific test. This may lead to false-negative results owing to a low frequency of the mutated allele [5]. Given the tendency for the AI algorithm to underestimate the tumor cell proportion, we can speculate that assessment by AI is less likely to produce a false-negative result.

The success rate was almost 100% for Oncomine DxTT testing and nearly 95% for AmoyDx PLC testing. All unsuccessful sample tests were a result of RNA analysis failure; there were no failures in the DNA analyses. Previous studies have reported a success rate of 75% to 93% for Oncomine DxTT tests [20,21,22,23,24,25] and 98.5% for AmoyDx PLC tests [26]; the present study had a slightly higher or similar success rate for the Oncomine DxTT and AmoyDx PLC tests, respectively. In an investigation of sample conditions for successful/unsuccessful Oncomine DxTT testing in Japan, tumor cell proportion, tumor cell count, and sample size were reported as influential factors [20,21]. In the present study, only five of the multiplex tests were unsuccessful, so it was difficult to conduct a meaningful evaluation of the relationship between sample condition and testing success rate. However, these samples tended to have smaller volumes, and the tumor cell proportion was determined to be <30% by both the Central Pathology Committee and AI algorithm in 5/5 samples (≥30% in 4/5 samples by local pathologists), indicating a similar trend to that previously reported [20,21].

There were no notable differences in the frequencies of driver gene alterations, with similar trends observed for the multiplex assays (Oncomine DxTT and AmoyDx PLC). The frequency of each alteration was generally similar to that reported in other studies conducted in Japan that evaluated these testing platforms [24,26], although we note that some alterations were not detected in any of our study samples. Considering the singleplex tests, 38.5% of samples tested positive for *EGFR* mutation with the cobas test, which was higher than that reported with the multiplex tests (Oncomine DxTT, 28.9%; AmoyDx PLC, 28.8%). However, direct comparisons are not appropriate given the small number of samples tested using the cobas method, and given that for each method, the percentage of positive samples was calculated for different populations. Prior to insurance coverage of multiplex tests in Japan, a systematic review of 33 Japanese studies reported that 45% of samples from patients with NSCLC of adenocarcinoma histology (2069/4619) were positive for *EGFR* mutation [27]. Additionally, the BRAVE study, which was conducted in a patient population similar to that of the present study, reported that 38.1% of patients (75/197) had *EGFR* mutations [28]. The results of the REVEAL cohort study were not always concordant between multiplex and singleplex tests, with a positive concordance rate of 93.5% (58/62) and a negative concordance rate of 98.6% (140/142) for *EGFR* mutation [29]. There are two main reasons for this discrepancy: tests using next-generation DNA sequencing, such as Oncomine DxTT, tend to be less sensitive than polymerase chain reaction-based testing, such as cobas, and there are slight differences in the types of mutations that can be detected using each method.

This study had some limitations that should be considered when interpreting the results. First, the true values for tumor cell proportions in the tissue samples were unknown, limiting the comparisons between groups. The total number of nucleated cells in a clinical sample can often reach several thousand to several hundred thousand, which is practically impossible for an individual to accurately differentiate from non-tumor cells and count. Thus, the true values for tumor cell proportion cannot be assessed. To address this, the ‘reference’ tumor cell proportion was assessed by the Central Pathology Committee, which was composed of three skilled specialists. The three specialists were in complete agreement (3/3 specialists), majority agreement (2/3 specialists), and no agreement for 7, 90, and 107 of the 204 patient tissue samples assessed, respectively. In the case of disagreement among the specialists, a consensus was reached following discussion. We think this approach lends a certain level of validity to the reference values. Second, for a task such as assessment of tumor cell proportion, it is assumed that intra-rater agreement will not be perfect with human assessment. However, this study did not collect data to evaluate this. In principle, the AI algorithm is fixed, and it can be assumed that there is no variability among multiple assessments of the same single tissue sample image. This highlights another potential advantage for the use of AI algorithms for this type of task. Third, given that nearly all the investigational sites were specialized centers (Designated Hospital for Cancer Genomic Medicine, *n* = 4; Cooperative Hospital for Cancer Genomic Medicine, *n* = 6), the results may not reflect the assessment of tumor cell proportion and the approaches to biomarker testing at non-specialist centers. Thus, the generalizability of the study findings to the wider Japanese population is limited. Finally, because this study did not collect any data for variant allele frequencies, it was difficult to explore the relevance between low tumor cell proportion in samples and potential false-negative results with Oncomine DxTT biomarker testing.

In conclusion, the agreement of the assessment of tumor cell proportions in prepared tissue samples between local pathologists and the Central Pathology Committee ranged from poor to moderate. A tendency for overestimation of tumor cell proportion by local pathologists was observed. The highest ICC identified among rater pairs was between the AI algorithm and the Central Pathology Committee. This trend appeared to be consistent across sampling method subgroups. The AI algorithm evaluated in this study may be a useful tool for assessing tumor cell proportion. Continued efforts are needed to ensure the accurate estimation of tumor cell proportion. However, although the use of AI algorithms in real-world practice may contribute to this process, the specific AI algorithm used in this study must be further evaluated and validated prior to implementation.

## Figures and Tables

**Table 1:** Baseline characteristics of patients in the full analysis set (*n* = 209) and tumor cell proportion analysis set (*n* = 204).

	Full Analysis Set *n* = 209	Tumor Cell Proportion Analysis Set *n* = 204
Age, median (range), y	70.0 (36.0–90.0)	70.0 (36.0–90.0)
Sex		
Male	136 (65.1)	131 (64.2)
Female	73 (34.9)	73 (35.8)
Smoking status		
Current	35 (16.7)	34 (16.7)
Former	117 (56.0)	113 (55.4)
Never	57 (27.3)	57 (27.9)
Histologic type		
Adenocarcinoma	189 (90.4)	184 (90.2)
Other	20 (9.6)	20 (9.8)
ECOG PS		
0	83 (39.7)	82 (40.2)
1	106 (50.7)	103 (50.5)
2	15 (7.2)	14 (6.9)
3	3 (1.4)	3 (1.5)
4	2 (1.0)	2 (1.0)
Clinical stage		
IIIB	3 (1.4)	3 (1.5)
IIIC	0	0
IVA	67 (32.1)	66 (32.4)
IVB	119 (56.9)	117 (57.4)
Post-operative recurrence	20 (9.6)	18 (8.8)

Data are *n* (%) unless otherwise stated. Abbreviation: ECOG PS, Eastern Cooperative Oncology Group performance status.

**Table 2:** Tissue sample collection and fixation conditions in the tumor cell proportion analysis set (*n* = 204).

	Tumor Cell Proportion Analysis Set *n* = 204
Sampling location, *n* (%)	
Primary tumor	153 (75.0)
Lymph node	38 (18.6)
Intrathoracic	32
Extrathoracic	6
Metastatic lesion	13 (6.4)
Bone	5
Brain	2
Lung	2
Pleural nodule	2
Liver	1
Adrenal gland	1
Sampling method, *n* (%)	
Surgical resection	28 (13.7)
Bronchoscope biopsy	157 (77.0)
EBUS-GS	48
EBUS-TBNA	43
Transbronchial lung biopsy	43
Endobronchial biopsy	23
Computed tomography-guided needle biopsy	16 (7.8)
Echo-guided needle biopsy	2 (1.0)
Other ^a^	1 (0.5)
Type of fixing solution, *n* (%)	
10% neutral buffered formalin solution	202 (99.0)
Other ^b^	2 (1.0)
Fixation time, *n* (%)	
<6 h	4 (2.0)
6–<12 h	14 (6.9)
12–<24 h	177 (86.8)
24–<48 h	7 (3.4)
48–72 h	1 (0.5)
Unknown	1 (0.5)
Thickness of FFPE sections, mean (SD), μm	5.00 (0.666)
Number of slides submitted for biomarker testing, median (range)	10.0 (4.0–30.0)
Estimated sample volume ^c^, median (range), mm^3^	1.39 (0.06–39.51)

^a^ Endoscopic ultrasound fine-needle aspiration. ^b^ 20% neutral buffered formalin solution. ^c^ Estimated sample volume = [thickness of FFPE sections] × [number of slides submitted for biomarker testing] × [surface area of section]. The surface area of the section was assessed using the artificial intelligence algorithm. Abbreviations: EBUS-GS, endobronchial ultrasound using a guide sheath; EBUS-TBNA, endobronchial ultrasound transbronchial needle aspiration; FFPE, formalin-fixed paraffin-embedded; SD, standard deviation.

**Table 3:** Summary of pathological features in H&E-stained slides assessed by the Central Pathology Committee (tumor cell proportion analysis set, *n* = 204).

	Tumor Cell Proportion Analysis Set *n* = 204
Inflammatory cells ^a^	
None or mild	37 (18.1)
Moderate	135 (66.2)
Severe	32 (15.7)
Fibrosis ^b^	
None or mild	142 (69.6)
Moderate	56 (27.5)
Severe	6 (2.9)
Mucus ^b^	
None or mild	199 (97.5)
Moderate	4 (2.0)
Severe	1 (0.5)
Necrosis ^b^	
None or mild	192 (94.1)
Moderate	8 (3.9)
Severe	4 (2.0)
Crush ^c^	
None or mild	182 (89.2)
Moderate	18 (8.8)
Severe	4 (2.0)

Data are *n* (%). Proportions were calculated using all analyzed areas as the denominator and the area affected by each pathological finding as the numerator. ^a^ None or mild, <10%; moderate, 10–70%; severe, >70%. ^b^ None or mild, <33%; moderate, 33–66%; severe, >66%. ^c^ None or mild, 0–<3 crushes that have a minor impact on the overall assessment; moderate, ≥3 crushes that have a minor impact on the overall assessment; severe, crushes that have a major impact on the overall assessment. Abbreviation: H&E, hematoxylin and eosin.

**Table 4:** Comparison of ICC values for tumor cell proportion assessment between each pair of raters by sampling method (tumor cell proportion analysis set, *n* = 204).

	Tumor Cell Proportion Analysis Set *n* = 204		
	Local Pathologists vs. Central Pathology Committee	AI Algorithm vs. Central Pathology Committee	AI Algorithm vs. Local Pathologists
Surgical resection (*n* = 28)	0.534 (0.217, 0.751)	0.642 (0.357, 0.817)	0.330 (0.000, 0.617)
Bronchoscope biopsy (*n* = 157)	0.603 (0.489, 0.697)	0.643 (0.500, 0.744)	0.485 (0.252, 0.644)
Computed tomography-guided needle biopsy (*n* = 16)	0.558 (0.087, 0.821)	0.621 (0.205, 0.848)	0.415 (0.000, 0.750)

Data are ICC (95% CI). ICC values in samples obtained by echo-guided needle biopsy (*n* = 2) and others (*n* = 1) are not presented because of the small sample sizes.

**Table 5:** Categories of absolute differences in tumor cell proportion assessment values between each pair of raters (tumor cell proportion analysis set, *n* = 204).

	Tumor Cell Proportion Analysis Set *n* = 204		
	No or Slight Difference	Moderate Difference	Considerable Difference
Local pathologists vs. Central Pathology Committee	125 (61.3)	38 (18.6)	41 (20.1)
AI algorithm vs. Central Pathology Committee	134 (65.7)	35 (17.2)	35 (17.2)
AI algorithm vs. local pathologists	115 (56.4)	44 (21.6)	45 (22.1)

Data are *n* (%).

**Table 6:** Proportions of individual gene alterations according to the biomarker testing method used in the full analysis set (*n* = 209).

	Oncomine™ Dx Target Test Multi CDx System	AmoyDx^®^ Pan Lung Cancer PCR Panel	cobas^®^ EGFR Mutation Test	Archer^®^MET Companion Diagnostic System
*EGFR* mutation	35/121 (28.9)	21/73 (28.8)	5/13 (38.5)	-
*ALK* fusion	2/120 (1.7)	3/69 (4.3)	-	-
*ROS1* fusion	0/120 (0.0)	1/69 (1.4)	-	-
*BRAF* mutation	0/121 (0.0)	2/73 (2.7)	-	-
*RET* fusion	1/120 (0.8)	0/64 (0.0)	-	-
*MET* exon 14 skipping	7/114 (6.1)	1/69 (1.4)	-	0/2 (0.0)
*KRAS* mutation	6/115 (5.2)	6/68 (8.8)	-	-
*HER2* mutation	2/115 (1.7)	1/65 (1.5)	-	-
*NTRK* fusion	0/114 (0.0)	0/64 (0.0)	-	-

Data are *n1/n* (%). *n1*, number of positive samples; *n*, (number of positive samples) + (number of negative samples). Abbreviations: *HER2*, human epidermal growth factor receptor-2; *KRAS*, v-Ki-ras2 Kirsten rat sarcoma viral oncogene homolog; *NTRK*, neurotrophic tyrosine receptor kinase.

## Data Availability

The data underlying the findings described in this manuscript may be obtained in accordance with AstraZeneca’s data sharing policy described at https://astrazenecagrouptrials.pharmacm.com/st/submission/disclosure (accessed on 18 April 2025). Data for studies directly listed on Vivli can be requested through Vivli at www.vivli.org (accessed on 18 April 2025). Data for studies not listed on Vivli could be requested through Vivli at https://vivli.org/members/enquiries-about-studies-not-listed-on-the-vivli-platform/ (accessed on 18 April 2025). The AstraZeneca Vivli member page is also available, outlining further details: https://vivli.org/ourmember/astrazeneca/ (accessed on 18 April 2025).

## Abbreviations

The following abbreviations are used in this manuscript:

AI	Artificial intelligence
ALK	Anaplastic lymphoma kinase
AmoyDx PLC	AmoyDx^®^ Pan Lung Cancer PCR Panel
BRAF	V-raf murine sarcoma viral oncogene homolog B1
CI	Confidence interval
CT	Computed tomography
EBUS-GS	Endobronchial ultrasound using a guide sheath
EBUS-TBNA	Endobronchial ultrasound transbronchial needle aspiration
ECOG PS	Eastern Cooperative Oncology Group performance status
EGFR	Epidermal growth factor receptor
FAS	Full analysis set
FFPE	Formalin-fixed paraffin-embedded
H&E	Hematoxylin and eosin
HER2	Human epidermal growth factor receptor-2
ICC	Intraclass correlation coefficient
KRAS	V-Ki-ras2 Kirsten rat sarcoma viral oncogene homolog
MET	Mesenchymal–epithelial transition
NSCLC	Non-small cell lung cancer
NTRK	Neurotrophic tyrosine receptor kinase
Oncomine DxTT	Oncomine^™^ Dx Target Test Multi CDx System
RET	Rearranged during transfection
ROS1	ROS proto-oncogene 1
SD	Standard deviation
TCP	Tumor cell proportion analysis set

## References

[1] NIH National Cancer Institute Non-Small Cell Lung Cancer Treatment (PDQ^®^)—Health Professional Version. https://www.cancer.gov/types/lung/hp/non-small-cell-lung-treatment-pdq#_484856_toc.

[2] Japan Lung Cancer Society Guidelines for Diagnosis and Treatment of Lung Cancer—Including Malignant Pleural Mesothelioma and Thymic Tumors 2024. https://www.haigan.gr.jp/publication/guideline/examination/2024/.

[3] Penault-Llorca F., Kerr K.M., Garrido P., Thunnissen E., Dequeker E., Normanno N., Patton S.J., Fairley J., Kapp J., de Ridder D. (2022). Expert Opinion on NSCLC Small Specimen Biomarker Testing—Part 2: Analysis, Reporting, and Quality Assessment. Virchows Arch..

[4] Hatanaka Y., Kuwata T., Morii E., Kanai Y., Ichikawa H., Kubo T., Hatanaka K.C., Sakai K., Nishio K., Fujii S. (2021). The Japanese Society of Pathology Practical Guidelines on the Handling of Pathological Tissue Samples for Cancer Genomic Medicine. Pathol. Int..

[5] The Biomarker Committee of The Japan Lung Cancer Society (2024). The Guidance for Biomarker Testing in Lung Cancer Patients. https://www.haigan.gr.jp/publication/guidance/inspection/.

[6] Smits A.J., Kummer J.A., de Bruin P.C., Bol M., van den Tweel J.G., Seldenrijk K.A., Willems S.M., Offerhaus G.J., de Weger R.A., van Diest P.J. (2014). The Estimation of Tumor Cell Percentage for Molecular Testing by Pathologists Is Not Accurate. Mod. Pathol..

[7] Viray H., Li K., Long T.A., Vasalos P., Bridge J.A., Jennings L.J., Halling K.C., Hameed M., Rimm D.L. (2013). A Prospective, Multi-Institutional Diagnostic Trial to Determine Pathologist Accuracy in Estimation of Percentage of Malignant Cells. Arch. Pathol. Lab. Med..

[8] Mikubo M., Seto K., Kitamura A., Nakaguro M., Hattori Y., Maeda N., Miyazaki T., Watanabe K., Murakami H., Tsukamoto T. (2020). Calculating the Tumor Nuclei Content for Comprehensive Cancer Panel Testing. J. Thorac. Oncol..

[9] Alowais S.A., Alghamdi S.S., Alsuhebany N., Alqahtani T., Alshaya A.I., Almohareb S.N., Aldairem A., Alrashed M., Bin Saleh K., Badreldin H.A. (2023). Revolutionizing Healthcare: The Role of Artificial Intelligence in Clinical Practice. BMC Med. Educ..

[10] Cosatto E., Gerard K., Graf H.P., Ogura M., Kiyuna T., Hatanaka K.C., Matsuno Y., Hatanaka Y., Tomaszewski J.E., Ward A.D. (2021). A Multi-Scale Conditional Deep Model for Tumor Cell Ratio Counting. Proceedings of the SPIE Medical Imaging 2021: Digital Pathology.

[11] Kiyuna T., Cosatto E., Hatanaka K.C., Yokose T., Tsuta K., Motoi N., Makita K., Shimizu A., Shinohara T., Suzuki A. (2024). Evaluating Cellularity Estimation Methods: Comparing AI Counting with Pathologists’ Visual Estimates. Diagnostics.

[12] The Japanese Society of Pathology, Japanese Society of Digital Pathology, Digital Pathology Technical Standard Review Committee (2018). Digital Pathology System Technical Standard for Pathological Diagnosis, 3rd Edition. https://pathology.or.jp/news/pdf/kijjun-181222.pdf.

[13] Ronneberger O., Fischer P., Brox T., Navab N., Hornegger J., Wells W., Frangi A. (2015). U-Net: Convolutional Networks for Biomedical Image Segmentation. Proceedings of the Medical Image Computing and Computer-Assisted Intervention—MICCAI 2015, Part III of the 18th International Conference.

[14] Bonett D.G. (2002). Sample Size Requirements for Estimating Intraclass Correlations with Desired Precision. Stat. Med..

[15] McGraw K.O., Wong S.P. (1996). Forming Inferences About Some Intraclass Correlation Coefficients. Psychol. Methods.

[16] Koo T.K., Li M.Y. (2016). A Guideline of Selecting and Reporting Intraclass Correlation Coefficients for Reliability Research. J. Chiropr. Med..

[17] Kazdal D., Rempel E., Oliveira C., Allgäuer M., Harms A., Singer K., Kohlwes E., Ormanns S., Fink L., Kriegsmann J. (2021). Conventional and Semi-Automatic Histopathological Analysis of Tumor Cell Content for Multigene Sequencing of Lung Adenocarcinoma. Transl. Lung Cancer Res..

[18] Carretero-Barrio I., Pijuan L., Illarramendi A., Curto D., López-Ríos F., Estébanez-Gallo Á., Castellvi J., Granados-Aparici S., Compañ-Quilis D., Noguera R. (2024). Concordance in the Estimation of Tumor Percentage in Non-Small Cell Lung Cancer Using Digital Pathology. Sci. Rep..

[19] Stankovic B., Bjørhovde H.A.K., Skarshaug R., Aamodt H., Frafjord A., Müller E., Hammarström C., Beraki K., Bækkevold E.S., Woldbæk P.R. (2019). Immune Cell Composition in Human Non-Small Cell Lung Cancer. Front. Immunol..

[20] Takeyasu Y., Yoshida T., Motoi N., Teishikata T., Tanaka M., Matsumoto Y., Shinno Y., Okuma Y., Goto Y., Horinouchi H. (2021). Feasibility of Next-Generation Sequencing (Oncomine™ DX Target Test) for the Screening of Oncogenic Mutations in Advanced Non-Small-Cell Lung Cancer Patients. Jpn. J. Clin. Oncol..

[21] Nemoto D., Yokose T., Katayama K., Murakami S., Kato T., Saito H., Suzuki M., Eriguchi D., Samejima J., Nagashima T. (2021). Tissue Surface Area and Tumor Cell Count Affect the Success Rate of the Oncomine Dx Target Test in the Analysis of Biopsy Tissue Samples. Thorac. Cancer.

[22] Murakami S., Yokose T., Nemoto D., Suzuki M., Usui R., Nakahara Y., Kondo T., Kato T., Saito H. (2021). Suitability of Bronchoscopic Biopsy Tissue Samples for Next-Generation Sequencing. Diagnostics.

[23] Ariyasu R., Uchibori K., Ninomiya H., Ogusu S., Tsugitomi R., Manabe R., Sakamaoto H., Tozuka T., Yoshida H., Amino Y. (2021). Feasibility of Next-Generation Sequencing Test for Patients with Advanced NSCLC in Clinical Practice. Thorac. Cancer.

[24] Sakata S., Otsubo K., Yoshida H., Ito K., Nakamura A., Teraoka S., Matsumoto N., Shiraishi Y., Haratani K., Tamiya M. (2022). Real-World Data on NGS Using the Oncomine DxTT for Detecting Genetic Alterations in Non-Small-Cell Lung Cancer: WJOG13019L. Cancer Sci..

[25] Iwama E., Yamamoto H., Okubo F., Ijichi K., Ibusuki R., Shiaraishi Y., Yoneshima Y., Tanaka K., Oda Y., Okamoto I. (2023). Evaluation of Appropriate Conditions for Oncomine DxTT Testing of FFPE Specimens for Driver Gene Alterations in Non-Small Cell Lung Cancer. Thorac. Cancer.

[26] Kunimasa K., Matsumoto S., Kawamura T., Inoue T., Tamiya M., Kanzaki R., Maniwa T., Okami J., Honma K., Goto K. (2023). Clinical Application of the AMOY 9-in-1 Panel to Lung Cancer Patients. Lung Cancer.

[27] Midha A., Dearden S., McCormack R. (2015). *EGFR* Mutation Incidence in Non-Small-Cell Lung Cancer of Adenocarcinoma Histology: A Systematic Review and Global Map by Ethnicity (mutMapII). Am. J. Cancer Res..

[28] Shimizu J., Masago K., Saito H., Nishino K., Kurata T., Itoh Y., Yoshimura Y., Yabuki Y., Dosaka-Akita H. (2020). Biomarker Testing for Personalized, First-Line Therapy in Advanced Nonsquamous Non-Small Cell Lung Cancer Patients in the Real World Setting in Japan: A Retrospective, Multicenter, Observational Study (the BRAVE Study). Ther. Adv. Med. Oncol..

[29] Sakamoto T., Matsubara T., Takahama T., Yokoyama T., Nakamura A., Tokito T., Okamoto T., Akamatsu H., Oki M., Sato Y. (2023). Biomarker Testing in Patients with Unresectable Advanced or Recurrent Non-Small Cell Lung Cancer. JAMA Netw. Open.

